# Bi-directional long short term memory-gated recurrent unit model for Amharic next word prediction

**DOI:** 10.1371/journal.pone.0273156

**Published:** 2022-08-18

**Authors:** Demeke Endalie, Getamesay Haile, Wondmagegn Taye

**Affiliations:** 1 Faculty of Computing and Informatics, Jimma Institute of Technology, Jimma, Ethiopia; 2 Faculty of Civil and Environmental Engineering, Jimma Institute of Technology, Jimma, Ethiopia; Fuzhou University, CHINA

## Abstract

The next word prediction is useful for the users and helps them to write more accurately and quickly. Next word prediction is vital for the Amharic Language since different characters can be written by pressing the same consonants along with different vowels, combinations of vowels, and special keys. As a result, we present a Bi-directional Long Short Term-Gated Recurrent Unit (BLST-GRU) network model for the prediction of the next word for the Amharic Language. We evaluate the proposed network model with 63,300 Amharic sentence and produces 78.6% accuracy. In addition, we have compared the proposed model with state-of-the-art models such as LSTM, GRU, and BLSTM. The experimental result shows, that the proposed network model produces a promising result.

## 1. Introduction

In today’s digital era, one of the most important activities for people who use computers and handheld electronic devices is data entering. Next word prediction during data entry enhances document creation by eliminating spelling errors and speeding up text entry for users with low spelling skills [1]. Next word prediction benefits people with severe motor and oral disabilities, on handwriting recognition, mobile phone, or pad texting. In addition, word sequence prediction can be used as input for a variety of natural language processing studies including speech recognition and handwritten recognition [2].

Now a day’s, the usage of computers and handheld devices is growing day to day in Ethiopia. A large number of people use the Amharic language to communicate. Amharic is a Semitic language of the Afro-Asiatic Language Group that is related to Hebrew, Arabic, and Syrian [3]. Amharic is the working language of the Federal Democratic Republic of Ethiopia (EFDR) [4]. With this in mind, having an alternative or assistive Amharic text entry system is useful to speed up text entry, and helps those needing alternative communication. Amharic is one of the Ethiopian languages categorized as under-resourced [5]. Currently, the Amharic language requires a plugin or software that corrects and speeds up the writing of books, articles, newspapers, and reports.

Next word prediction is the process of predicting the upcoming next word based on its context [6]. Word prediction has a great role in avoiding or reducing typing errors and the number of keystrokes. The next word can be predicted based on the probability of prior words and context. For instance, if the user is writing the phrase “እየሱስ ክርስቶስ” (Jesus Christ), the user will first write the word “እየሱስ” from the keyboard and will predict the next word from the probable words and the following list of the high-frequency word next to "እየሱስ " that appears in the word prediction window based on the training corpus and select "ክርስቶስ” when it is available.

In the paper [7], the authors studied how different sequence-to-sequence deep learning models perform in the task of generating new conversations between characters. A comprehensive comparison between these models, namely, LSTM, GRU, and Bi-directional Recurrent Neural Network (RNN) is presented. All the models are designed to learn the sequence of recurring characters from the input sequence. Each input sequence will contain, say "n" characters, and the corresponding targets will contain the same number of characters, except, they will be shifted one character to the right. The loss analysis shows that bi-directional RNN has the lowest loss and GRU has the highest. GRU works a little faster and bi-directional RNN has the longest execution time.

The paper [8], presented a stochastic model-based next word prediction for the Urdu language. The Hidden Markov model was implemented to predict the next state, while Unigram Model was also used to suggest the current state and the next hidden state, N-Gram Model was followed keeping N = 2. The tool is developed to implement this model for Urdu Language (UL) and tested by regular and new content writers to check their improvements in their typing speeds.

The paper [9], presented a next word prediction algorithm for automatic code completion. The authors used two key constituents, prediction of within-vocabulary words and prediction of identifiers. In terms of predicting within-vocabulary words, a neural language model based on an LSTM network was proposed. Regarding the prediction of identifiers, a model based on a pointer network is proposed. For evaluation of the proposed method, source code accumulated in an online judge system is used. The results of the experiment demonstrate that the proposed method can predict both the next within-vocabulary word and the next identifier to a high degree of accuracy.

In [10], the authors presented an LSTM model to predict the next word(s) given a set of current words to the user for the Assamese language. They handle the problem of words which has multiple synonyms, by storing the transcripted Assamese language according to the International Phonetic Association (IPA). Their model goes through their dataset of the transcripted Assamese words and predicts the next word using LSTM with an accuracy of 88.20%. The result shows LSTM is prominently effective in next word prediction even for under-resourced language.

In the work of [11], the authors proposed a Multi-window Convolution Neural Network and Residual-connected Minimal Gated Unit (MCNN-ReMGU) network model for natural language word prediction. The residual-connected processing of the MGU network solves the problem of vanishing gradient and network degradation. Their experimental results on the Penn Treebank and WikiText-2 datasets show that the proposed method has an advantage in word prediction applications.

The paper [12], presented the application of long-term memory- recursive network in the generation of text sequence in real-time conditions by predicting a single data point at a time. When going through the text, people accept every word based on their interpretation of the preceding words. People can understand and respond, indicating that our thoughts tend to be ceaseless. Typical neural networks have a major drawback in that they shrink during such assumptions. Their Recursive neural networks provided a solution to this problem.

For the past two decades, LSTM and BLSTM have been widely used for next word prediction in different languages. BLSTM learns the bi-directional dependence between terms or words. However, it results in a more complex network model that takes longer to train [13]. Whereas the LSTM model learns the sequence of words in a sentence in just one way, either forward or backward. Due to this, the model does not properly understand the sequence [14]. As a result, the purpose of this study is to provide deep learning algorithms: the BLSTM-GRU model for predicting the next Amharic word. GRU was utilized as a second layer of BLSTM in the proposed model to minimize network complexity.

The main contributions of this study according to automated Amharic next word prediction are the following.

1. Develop an Amharic sentence dataset for future research.
2. A hybrid network model was designed based on the BLSTM-GRU cell unit for Amharic next word prediction.
3. The proposed method efficiency is compared to various deep learning-based next word prediction models.

The rest of the paper is organized as follows. The materials and methods describe the proposed approach to predict the next word for the Amharic language and the prepared dataset. Results and discussions present experimental setups used in the experimentation process. The conclusion consists of the main result and future work of the study.

## 2. Materials and methods

**Fig 1:**
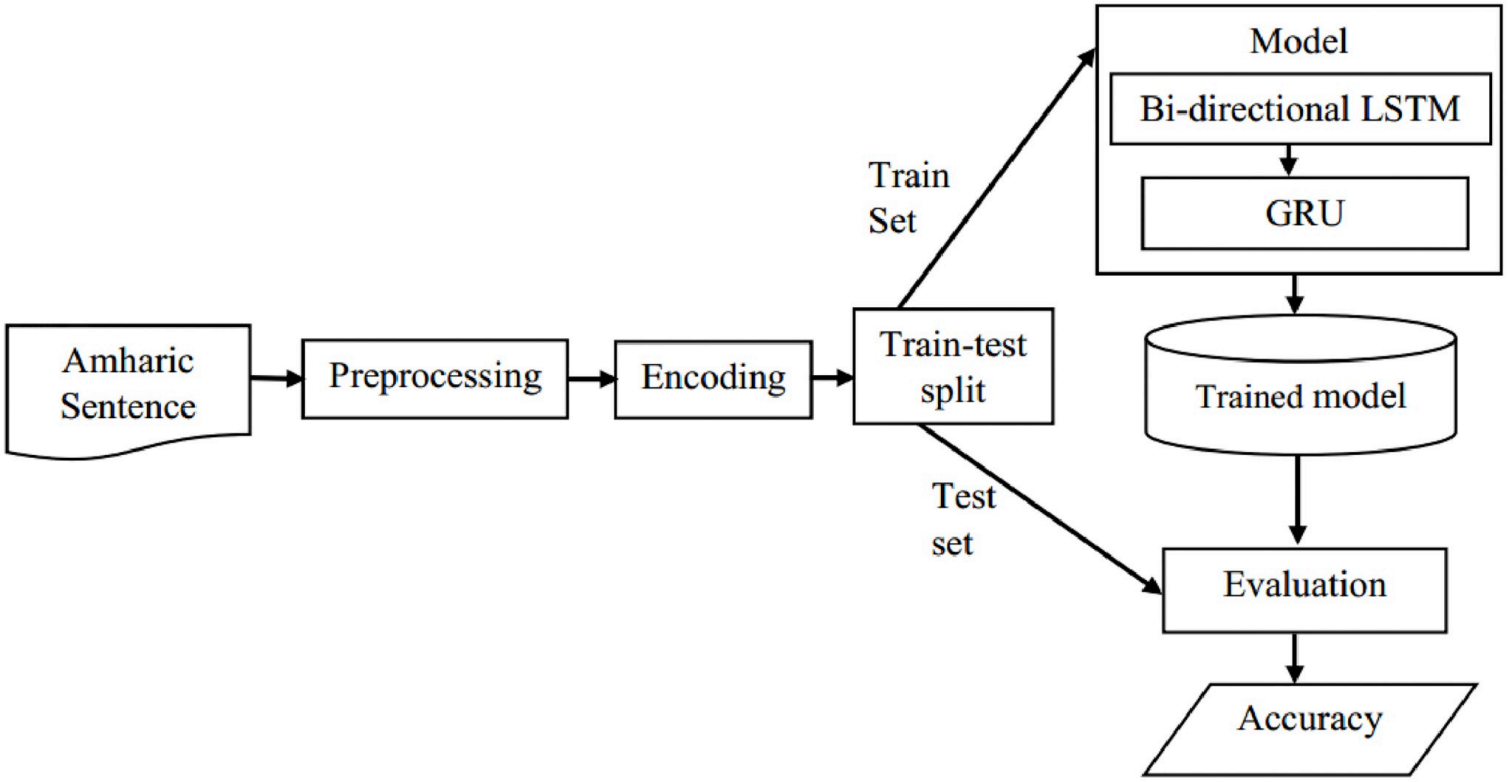
Proposed next word prediction model.

The proposed next word prediction model is built on a set of Amharic sentences that have different lengths (number of tokens). We use padding zero at the end of short sentences to make all the sentences the same length. The encoded sentence is then given to the BLSTM-GRU model’s input layer. The hyperparameters of the proposed model are determined through experiments. The model learns the order of words from the training sentences. The model can then be utilized in applications that write Amharic words after it has been trained and saved. This helps to save time and prevent typing errors. For example, in an Amharic statement, "እየሱስ ክርስቶስ ወደ እዚህ ምድር መጣ።" meaning "Jesus Christ came to this earth". The algorithm pops the following word "ክርስቶስ" while the writer inputs the word "እየሱስ". It saves time by not having to type each character "ክ", "ር", "ስ", "ቶ" and "ስ". The proposed next prediction model’s modules and components are presented in Fig 1.

### 2.1. Dataset description

There is no freely accessible Amharic sentence corpus for broad research purposes. As a consequence, we gather and compile the Amharic sentence dataset from the Amharic bible. The dataset obtained consists of 42,908 sentences for training, 13,850 sentences for validation, and 6,542 sentences for testing the model with varying lengths. The dataset contains sentences with a maximum length of 50 tokens and a minimum length of two tokens. After collecting, text cleaning is a mandatory step when we are working with text in Natural Language Processing (NLP). As a result, we apply the following preprocessing module to the dataset.

#### Punctuation and number removal

Since the main aim of this study is to predict the next word of a given term, we need to remove those tokens which consist of a number, punctuation marks, and token which contains non-Amharic characters. We used a regular expression to eliminate them from the dataset.

#### Tokenization

We utilized it to partition primary words from the next word and offer support to the BLSTM to observe word sequence in reverse and forward directions. For this purpose, we used a Python Natural Language Toolkit.

#### Integer encoding

Integer encoding consists of replacing the categories with digits from one to the number of distinct categories. We used integer encoding to convert each word to numerical data. We chose this method since it is very appropriate for a dataset with a larger size, straightforward to implement, and does not expand the feature space [15].

### 2.2. BLSTM

Bi-directional long-short term memory (BLSTM) is the method of making any neural network have the arrangement of data in both backward and forward directions [16, 17]. BLSTM utilizes most of the data by going the time-step in both directions. In bidirectional, our input flows in two directions, making a BLSTM different from the regular LSTM. With the regular LSTM, we can make input flow in one direction, either backward or forward. However, in bi-directional, we can make the input flow in both directions to preserve the future and the past information.

### 2.3. GRU

GRU is a simplified version of the LSTM recurrent neural network model [18, 19]. GRU uses only one state vector and two gate vectors, reset gate and update gate. The gated recurrent unit performs tasks of natural language processing, speech signal modeling, and music modeling like that of LSTM. The GRU model has been compared to LSTM and has been found to outperform LSTM when dealing with smaller datasets. In GRU, the input and forget gates are combined and controlled by one gate. This combination of gates makes GRU simpler than LSTM [20].

### 2.4. BLSTM-GRU model

The BLSTM learns the dependency between sequenced data in a Bi-directional manner, which is important to learn relevant features of the data in each time step. We add a dropout layer to the deep learning model to avoid overfitting [21]. This layer randomly drops out by a certain number of neurons to improve the network generalization. The second layer of the proposed network is the GRU cell unit. It is a special type of RNN with a fewer number of gates, which helps us to minimize the complexity of the network model [22].

**Fig 2:**
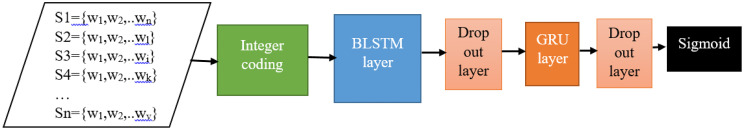
Proposed BLSTM-GRU network model for Amharic next word prediction.

The general structure of the proposed network architecture is depicted in Fig 2. The suggested network model’s input layer is a sequence of Amharic sentences. The input layer is used to feed the sequenced Amharic text to the network model, which is then processed by the embedding layer. To prevent overfitting, a bidirectional LSTM followed by a dropout layer is used. BLSTM assists in learning the relationship discovered between words by watching in both forward and backward directions. The suggested model’s second layer is a GRU cell unit that may extract contextual features at a lower computational cost.

The proposed model training algorithm can be described by the following steps.

Algorithm: Proposed BLSTM-GRU Model

Start

 1. *Input*: Amharic sentence sequence

 2. *Output*: next word of the given sequence

 3. BLSTM layer (hidden size, batch size)

 4. Dropout layer

 5. GRU layer (hidden size, batch size)

 6. Dropout layer

 7. Sigmoid activation function

End

### 2.5. Activation function

An activation function is a function that is added to an artificial neural network to help the network learn complex patterns in the data. An activation function is at the end deciding what is fired to the output layer. We used sigmoid as an activation function. The main reason why we use the sigmoid function is that the proposed model predicts probability as an output [23].

## 3. Results and discussion

### 3.1. Experimental setups

All experiments are carried out in a Windows 10 environment on a machine equipped with a Core i7 processor and 32 GB of RAM. The overall environment where the experiments are performed is illustrated in Table 1 below.

**Table 1:** System equipment.

Component	Description
CPU	Core i7
Language	Python
Operating system	Window 10
System type	64-bit
Memory	32 GB

### 3.2. Model training

In this phase, the main task is training the proposed network by using encoded Amharic sentence data. The proposed network model was implemented using Python deep learning library. All the hyperparameters of the proposed model were determined through experiments. The proposed BLSTM-GRU network model’s parameters are set as shown in Table 2.

**Table 2:** Parameter setting to train the proposed network model.

Parameters	Value
Number of neurons	256
Dropout	0.5
Optimizer	Adam
Batch size	64
Learning rate	0.001
Epoch	1000

**Fig 3:**
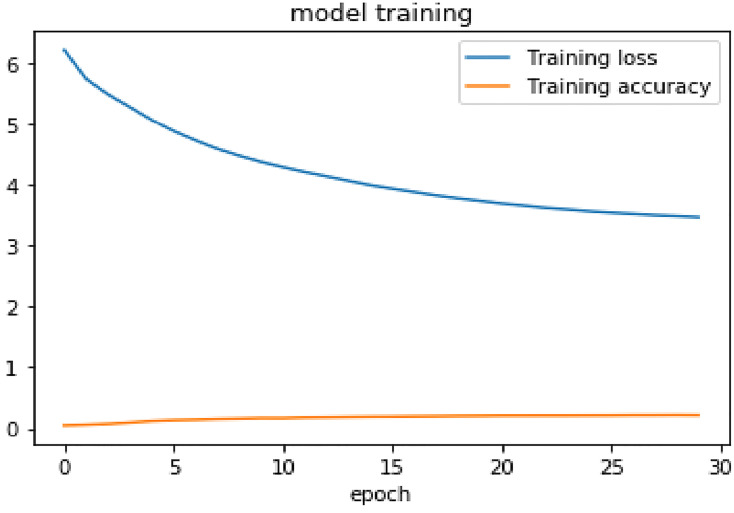
Training loss and training accuracy for 30 epoch.

Sample training categorical cross-entropy loss of the proposed model for the first 30 epochs is depicted as shown in Fig 3 below.

**Fig 4:**
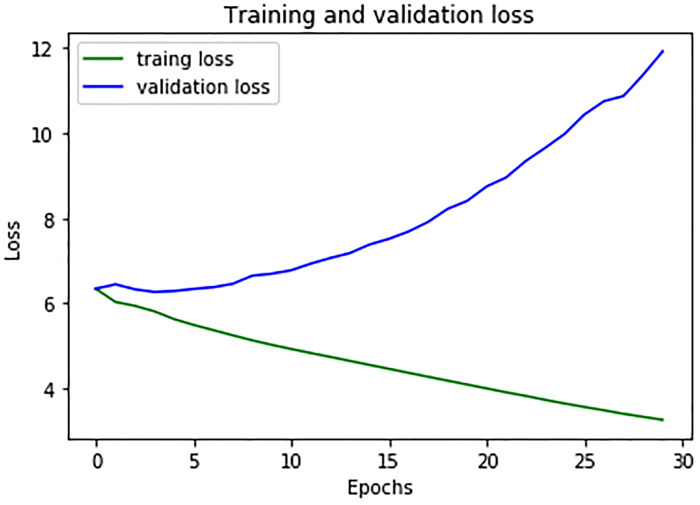
Training and validation loss of the model for 30 epoch.

We used 12,563 different Amharic sentences to validate the learning process of the proposed model. The model’s training and validation loss is projected in Fig 4 below.

### 3.3. Performance evaluation

Based on the device’s and parameter values stated in Tables 1 and 2 above, the overall performance of the proposed BLSTM-GRU network model is evaluated with a testing dataset and produces 78.6% prediction accuracy. The result of the proposed model evaluation on a testing dataset is displayed in Table 3. The performance of the model is evaluated in phrases of categorical cross-entropy loss and accuracy.

**Table 3:** Model evaluation.

Loss	Accuracy
0.4732	0.7859

We also evaluate the proposed BLSTM-GRU next word prediction model’s performance with an Amharic sentence that is not found in either the training or testing dataset. First, we load Pickle-formatted tokenized Amharic sentence file. Then, in the same directory as the source code, we load our proposed next word predictor model. Following this, we used the saved model to predict the input sentence. The Amharic word predicted by the provided phrase or sentence is shown in Fig 4 below.

**Fig 5:**
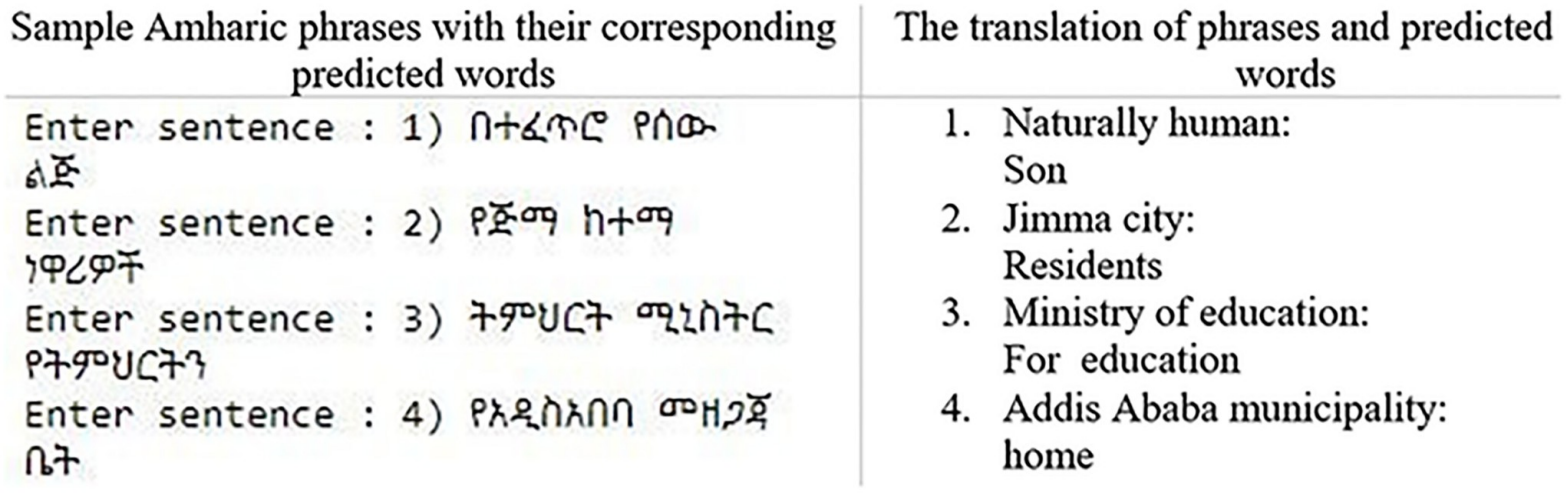
Sample predicted Amharic words from the provided phrase or sentence.

The result in Fig 5 shows, the predicted words are acceptable in Amharic writing system. Therefore the proposed BLSTM-GRU model can be embedded in different applications that are used for typing Amharic texts.

### 3.4 Model comparison

The overall performance of the proposed network model is compared with the state-of-the-art next word prediction models along with LSTM, GRU, and BLSTM models on the Amharic sentence dataset. The experimental result shows the proposed model improves next word prediction accuracy by 2.5% than the one which produces excellent from the remaining RNN models i.e., BLSTM. The evaluation result of the state-of-the-art next word prediction techniques with the proposed model is depicted in Table 4.

**Table 4:** Comparison of the proposed model with LSTM, GRU, and BLSTM.

Accuracy	LSTM	GRU	BLSTM	BLSTM-GRU
	75.02%	73.5%	76.1%	78.6%

Similarly to prediction accuracy, we compared the proposed BLSTM-GRU network model with LSTM, GRU, and BLSTM with the time required to finish the training. As a result, the proposed model outperforms LSTM and BLSTM models in terms of training time. Fig 4 below depicts the comparisons of the time required to train LSTM, GRU, BLSTM, and BLSTM-GRU.

**Fig 6:**
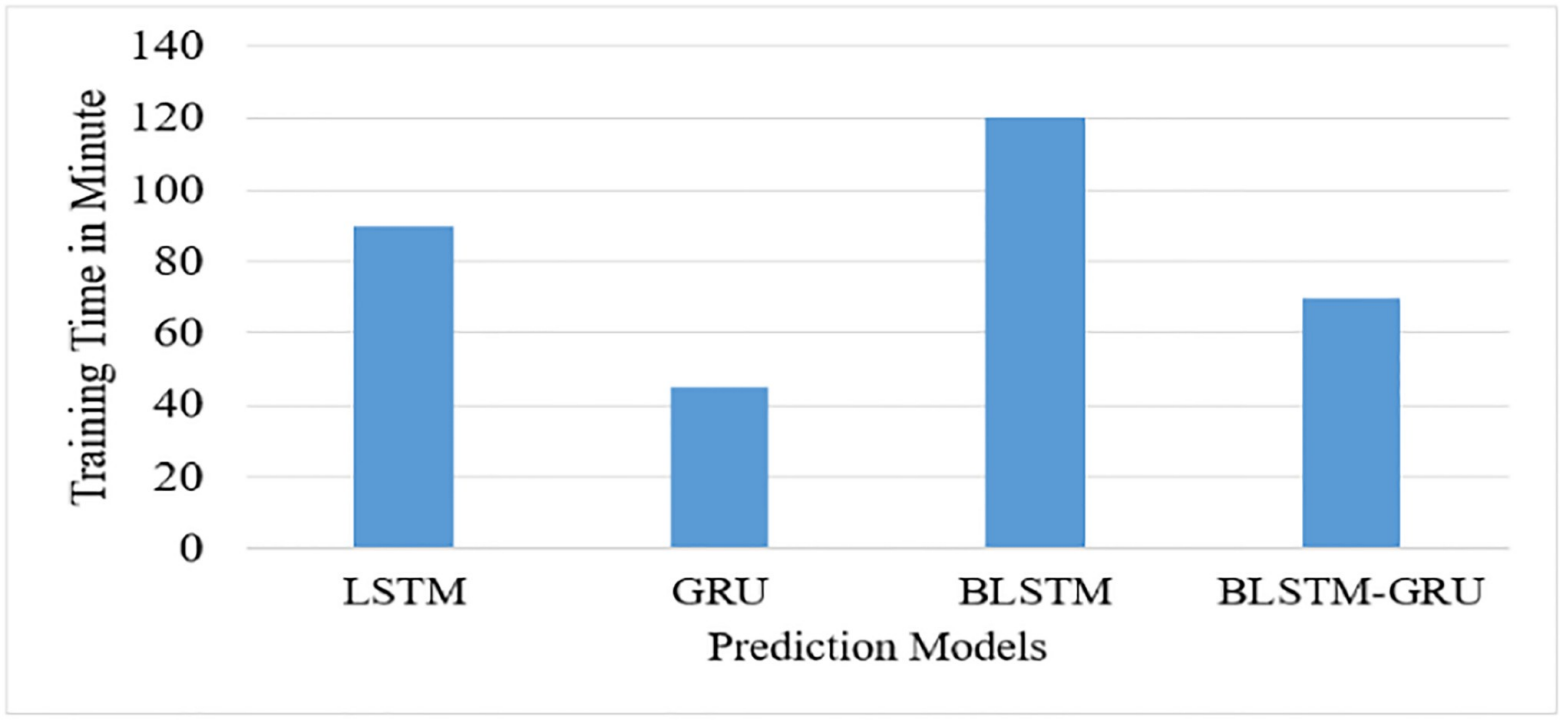
Comparison of the proposed model with LSTM, GRU, and BLSTM with training time.

As the result shown in Fig 6 above, the proposed predictive network model uses GRU in combination with BLSTM to minimize execution time. In general, the proposed network model compromises prediction accuracy and execution time by balancing the shortcomings of one model with the strengths of another. The model is tested with word-level predictions, not character sequence predictions.

## 4. Conclusion

This paper proposed the BLSTM-GRU network model to predict the next word for the Amharic writing system. The BLSTM showed a better understanding of the relationship between words by learning in a bi-directional manner. The GRU cell unit deploys an update and reset gate, which is more efficient than the conventional LSTM model. Different experiments were conducted to evaluate the performance of the proposed next word prediction model and to compare it with the state-of-the-art methods. The result shows that the proposed next word prediction network model (BLSTM-GRU) yielded a promising result. The proposed method can predict the next word in more complex sentences, in which the relationships and dependencies are more complex to discover. Future improvement should be focused on trying the proposed network model on the larger Amharic sentence dataset from different domains.

## References

[1] Selvi Kanimozhi, Ramya S., "Recurrent Neural Network based Models for Word Prediction," *International Journal of Recent Technology and Engineering (IJRTE)*, vol. 8, no. 4, pp. 7433–7437, 2019.

[2] Nadkarni Prakash M, Ohno-Machado Lucila, Chapman Wendy W, "Natural language processing: an introduction," *J Am Med Inform Assoc*., vol. 8, no. 5, p. 544–551, 2011. doi: 10.1136/amiajnl-2011-000464 21846786 PMC3168328

[3] Demeke Girma A., "The Ethio-Semitic Languages (Re-examining the Classification)," *Journal of Ethiopian Studies*, vol. 34, no. 2, pp. 57–93, 2001.

[4] Salawu Abiodun, Aseres Asemahagn, "Language policy, ideologies, power and the Ethiopian media," *South African Journal for Communication Theory and Research*, vol. 41, no. 1, pp. 71–89, 2015.

[5] Gereme Fantahun, Zhu William, Ayall Tewodros, Alemu Dagmawi, "Combating Fake News in “Low-Resource” Languages: Amharic Fake News Detection Accompanied by Resource Crafting," *Information*, vol. 12, no. 1, pp. 1–20, 2021.

[6] Shakhovska Khrystyna, Dumyn Iryna, Kryvinska Natalia, Kagita Mohan Krishna, "An Approach for a Next-Word Prediction for Ukrainian Language," *Wireless Communications and Mobile Computing*, vol. 2021, pp. 1–9, 2021.35573891

[7] Sanidhya Mangal, Poorva Joshi, Rahul Modak, LSTM vs. GRU vs. Bidirectional RNN for script generation, 2019.

[8] Hassan Muhammad, Saeed Muhammad, Nawaz Ali, Ahsan Kamran, Jabeen Sehar, Siddiqui Farhan Ahmed, et al, "Effective Word Prediction in Urdu Language Using Stochastic Model," *Sukkur IBA Journal of Computing and Mathematical Sciences*, vol. 2, no. 2, pp. 38–46, 2018.

[9] Terada Kenta and Watanobe Yutaka, "Code completion for programming education based on deep learning," *Int*. *J*. *Computational Intelligence Studies*, vol. 10, no. 2–3, pp. 109–114, 2021.

[10] Pratim Barman Partha, Boruah Abhijit, "A RNN based Approach for next word prediction in Assamese Phonetic Transcription," *Procedia Computer Science*, vol. 43, pp. 117–123, 2018.

[11] Yang Jingyun, Wang Hengjun, Guo Kexiang, "Natural Language Word Prediction Model Based on Multi-Window Convolution and Residual Network," *IEEE Access*, vol. 8, pp. 188036–188043, 2020.

[12] Khare Akash, Gupta Anjali, Mittal Anirudh and Jyoti Amrita, "Text Sequence Prediction Using Recurrent Neural Network," *Advances and Applications in Mathematical Sciences*, vol. 20, no. 3, pp. 377–382, 2021.

[13] Abduljabbar Rusul L., Dia Hussein, Tsai Pei-Wei, "Unidirectional and Bidirectional LSTM Models for Short-Term Traffic Prediction," *Journal of Advanced Transportation*, vol. 2021, pp. 1–16, 2021.

[14] Luigi Cerone Giacinto, Marco Knaflitz, Valentina Agostini, "Long short-term memory (LSTM) recurrent neural network for muscle activity detection," *Journal of NeuroEngineering and Rehabilitation*, vol. 18, no. 1, pp. 153–168, 2021. 34674720 10.1186/s12984-021-00945-w PMC8532313

[15] Chow Kevin, Tzamarias Dion Eustathios Olivier, Hernández-Cabronero Miguel, Blanes Ian Serra-Sagristà Joan, "Analysis of Variable-Length Codes for Integer Encoding in Hyperspectral Data Compression with the k2-Raster Compact Data Structure," *Remote sensing*, vol. 12, no. 12, p. 1983, 2020.

[16] Patel Suramya, Gite Shilpa, "Bi-directional Long Short-Term Memory with Convolutional Neural Network Approach for Image Captioning," *International Journal of Current Engineering and Technology*, vol. 7, no. 6, pp. 1968–1972, 2017.

[17] Maddu Rajesh; Vanga Abhishek Reddy; Sajja Jashwanth Kumar; Basha Ghouse; Shaik Rehana, "Prediction of land surface temperature of major coastal cities of India using bidirectional LSTM neural networks," *Journal of Water and Climate Change*, vol. 12, no. 8, p. Journal of Water and Climate Change, 2021.

[18] Mateus Balduíno César, Mendes Mateus, Farinha José Torres, Assis Rui, Cardoso António Marques, "Comparing LSTM and GRU Models to Predict the Condition of a Pulp Paper Press," *Energies*, vol. 14, pp. 6958–6979, 2021.

[19] Bhuvaneswari A., Timothy Jones Thomas J., Kesavan P., "Embedded Bi-directional GRU and LSTM Learning Model to Predict Disasterson Twitter Data," *Procedia Computer Science*, vol. 165, pp. 511–516, 2019.

[20] Ali Jaber Almalki, Pawel Wocjan, "Forecasting Method based upon GRU-based Deep Learning Model," in 2020 *International Conference on Computational Science and Computational Intelligence (CSCI)*, Las Vegas, NV, USA, 2020.

[21] Srivastava Nitish, Hinton Geoffrey, Krizhevsky Alex, Sutskever Ilya, Salakhutdinov Ruslan, "Dropout: A Simple Way to Prevent Neural Networks from Overfitting," *Journal of Machine Learning Research*, vol. 15, no. 1, pp. 1929–1958, 2014.

[22] Apaydin Halit, Feizi Hajar, Sattari Mohammad Taghi, Colak Muslume Sevba, Shamshirband Shahaboddin, Chau Kwok-Wing, "Comparative Analysis of Recurrent Neural Network Architectures for Reservoir Inflow Forecasting," *water*, vol. 12, no. 1500, pp. 1–18, 2020.

[23] Yuen Brosnan, Hoang Minh Tu, Dong Xiaodai & Lu Tao, "Universal activation function for machine learning," *Scientific Reports*, vol. 11, no. 1, p. 18757, 2021. doi: 10.1038/s41598-021-96723-8 34548504 PMC8455573

